# Nocturnal Glycemic Control with New Insulin Glargine 300 U/mL

**DOI:** 10.1155/2019/8587265

**Published:** 2019-06-26

**Authors:** Neng Chun Yu

**Affiliations:** Neng-Chun Diabetes Clinic, No. 491, Guangrong Rd., Luodong Township, Yilan County 265, Yilan, Taiwan

## Abstract

Insulin glargine 300 U/mL (Gla-300) is a new generation basal insulin product that has been demonstrated to have more stable pharmacokinetic and pharmacodynamic characteristics than insulin glargine 100 U/mL (Gla-100). To evaluate the real-world benefits of Gla-300 in reducing nocturnal fluctuations in blood glucose levels and nocturnal hypoglycemia, 10 Taiwanese patients using Gla-100 for insulin therapy were switched to Gla-300 and continuous glucose monitoring (CGM) was applied at nighttime to monitor changes to nocturnal glycemic variability parameters. Glycemic variability parameters measured to assess between- and within-night glycemic variability included mean 6-hour nocturnal (00:00–6:00 AM) glucose levels, standard deviation (SD), and coefficient of variance (CV) of mean nocturnal glucose levels and mean glucose excursion (MAGE). In this study, Gla-300 demonstrated comparable glycemic efficacy to Gla-100 and the potential to further reduce nocturnal hypoglycemia risk. Overall, nocturnal glycemic variability parameters measured during the Gla-300 treatment period were numerically smaller than those measured during the Gla-100 treatment phase although statistical significance was not reached. In terms of within-night glucose management, SD and CV values of mean nocturnal glucose levels were found to be statistically lower during the Gla-300 treatment phase than the Gla-100 treatment phase on nights individuals displayed normal blood glucose level readings at the beginning of the night. In summary, this study represents the first of its kind from Taiwan to evaluate the real-world clinical benefits of switching Taiwanese diabetes patients from Gla-100 to Gla-300 insulin therapy in reducing nighttime glucose variability by means of CGM.

## 1. Introduction

Insulin therapy is recommended by clinical guidelines as the primary strategy for the management of type 1 diabetes (T1DM) and as the second-line therapy for type 2 diabetes (T2DM) [1–3]. Nonetheless, while a number of advances have been made to improve glycemic control with long-acting insulin products, hypoglycemia, in particular, nocturnal hypoglycemia and its associated adverse health consequences, remains a constant concern for clinicians when prescribing and managing patients with basal insulins [4–6].

Gla-300 is a new long-acting insulin product delivering the same unit amount of insulin glargine in one-third of the volume compared to Gla-100 and provides even greater glycemic control and hypoglycemia-reducing effects than Gla-100 through better mimicry of the physiological profile of endogenous basal insulin secretion during periods of fasting, between meals, and sleep [7–9]. In studies comparing the pharmacokinetic/pharmacodynamic (PK/PD) characteristics between Gla-100 and Gla-300, at equivalent doses, Gla-300 is demonstrated to consistently produce flatter, more evenly distributed and sustained insulin concentration and plasma glucose concentration-time curves than Gla-100 [10, 11]. The improved PK/PD properties of Gla-300, compared with Gla-100 at matching drug doses, are attributed to the smaller surface area of the subcutaneous depots formed by Gla-300 [10, 11]. The smaller drug depots allow for a slower dissolution and more gradual release of glargine which results in the achievement of less variable glucose profiles that can be sustained in excess of 24 hours [10–13]. In a series of 6 noninferiority phase 3 trials, the EDITION trials, the clinical benefits associated with the improved PK/PD profiles of Gla-300 over Gla-100 in hemoglobin A1c (HbA1_c_), and fasting plasma glucose management have been demonstrated in several cohorts of T1DM and T2DM patient populations. Consistently, Gla-300 demonstrated comparable glycemic control as Gla-100 while significantly reducing the occurrence of nocturnal hypoglycemia events [14–19].

While Gla-300 has been approved in Taiwan, to our knowledge, the real-world benefits of switching Taiwanese diabetics from Gla-100 to Gla-300 on reducing nocturnal glucose variability and nocturnal hypoglycemia have not been previously evaluated. For this reason, in this case-series study, the potential real-world benefits of switching Taiwanese diabetes patients (T1DM and T2DM) from Gla-100 to Gla-300 for basal insulin therapy to minimize nocturnal glucose variability were investigated through continuous glucose monitoring (CGM).

## 2. Method

### 2.1. Study Design

In this case-series study, two rounds of nocturnal CGM were performed in a total of 10 diabetes patients who visited the clinic between April 2016 and August 2016 when they were being clinically switched from Gla-100 (Lantus, Sanofi, Paris, France) to Gla-300 (Toujeo, Sanofi, Paris, France) for basal insulin therapy. 8 patients were with T1DM, and 2 patients were with T2DM. However, in the clinic, patients continued to perform self-injections. The 1^st^ round of nocturnal CGM was performed prior to the switch to record variability in nocturnal glycemic parameters under Gla-100 therapy. The 2^nd^ round of nocturnal CGM was performed 1 week after patients' switched treatment to ensure steady-state control with Gla-300 was achieved. Unless patients' 1^st^ round nocturnal CGM traces indicated they required insulin dose adjustments, patients were prescribed Gla-300 at the same insulin dose used during the Gla-100 treatment period. For patients that required dose adjustment prior to switching to Gla-300, the dose adjustment algorithm for Gla-100 was used to calculate the required new dose and converted to Gla-300 equivalent doses. In total, 57 and 55 nights worth of CGM traces were recorded among the 10 participants during the Gla-100 and Gla-300 treatment phases, respectively.

CGM was performed using the iPro™2 professional CGM system (Medtronic, Taipei City, Taiwan). Nocturnal interstitial glucose levels (herein referred simply as nocturnal glucose levels) were sampled at 5-minute intervals during the 6-hour monitoring period of 00:00–6:00 AM. All procedures implemented were performed in accordance with the Declaration of Helsinki and Good Clinical Practice guidelines. Signed informed consent was obtained from all participants.

### 2.2. Assessment of between- and within-Night Nocturnal Glycemic Variability

The following parameters of glycemic variability were calculated and compared between Gla-100 and Gla-300 treatment phases from the CGM traces: (1) mean nocturnal glucose level, (2) standard deviation (SD) and (3) coefficient of variance (CV) values of mean nocturnal glucose level, and (4) mean glucose excursion.

### 2.3. Statistical Analysis

Data in graphs are presented as mean ± SEM. Descriptive and categorical data presented in text and tables are expressed as mean ± SD and as percentages, respectively. The chi-square test and paired *t*-test were applied to test for statistical significance of differences between descriptive and categorical data, respectively. A *p* value <0.05 was considered significant in both tests. All statistical analyses were performed using GraphPad Prism version 7.00 statistical software package (GraphPad Software, La Jolla, CA).

## 3. Results

### 3.1. Study Population

Baseline characteristics of participants in the study with a mean age of 44 ± 17 were as follows: mean duration of diabetes of 10 ± 6.1 years, mean HbA1_c_ of 7.5 ± 1.2%, and gender ratio of male vs. female as 20% : 80%. The average amount of insulin units (U) administered for basal insulin therapy per patient was 12.5 ± 7.0 U and 12.2 ± 6.5 U in the Gla-100 and Gla-300 treatment phase, respectively. No significant difference was observed in the mean insulin dose used between Gla-100 and Gla-300 treatment phases.

### 3.2. Assessment of Nocturnal Glucose Variability by CGM

Calculated mean nocturnal (00:00–6:00 AM) glycemic variability parameters for each treatment period pooled across all individuals are presented in Table 1. Consistent with other Gla-100 to Gla-300 drug-switching CGM studies, the mean nocturnal glucose level achieved by individuals during the Gla-300 treatment phase was equivalent to that achieved during the Gla-100 therapy phase. The variability in mean nocturnal glucose levels (i.e., SD and CV values of mean nocturnal glucose levels) and mean amplitude of glucose excursions experienced by individuals between-nights was lower during the Gla-300 treatment period than the Gla-100 treatment period, albeit no significant differences in these metrics of glycemic variability between the two treatments were reached.

### 3.3. Frequency of Hypoglycemia, Normoglycemia, and Hyperglycemia at 00:00 AM

**Figure 1:**
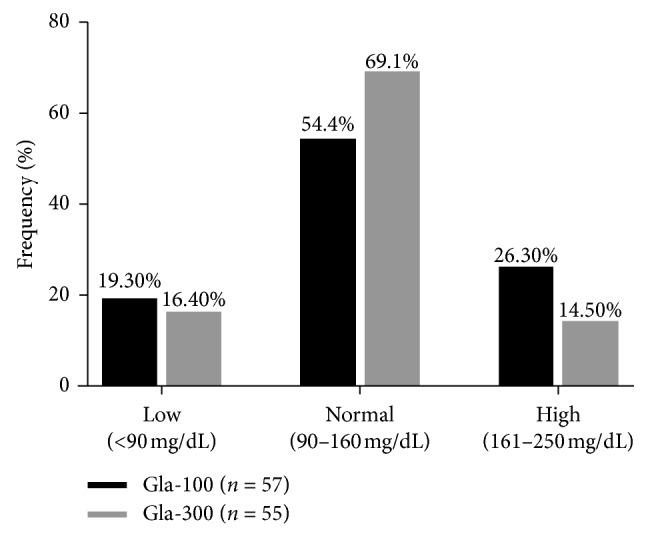
Cumulative frequency (%) of patients presenting with low (<90 mg/dL), normal (90-160 mg/dL), or high (161–250 mg/dL) nocturnal glucose levels at 00:00 AM on CGM nights.

The cumulative frequency of patients to present with low (<90 mg/dL), normal (90–160 mg/dL), or high (161–250 mg/dL) nocturnal glucose levels at 00:00 AM on CGM nights is presented in Figure 1. Normoglycemia at 00:00 AM was recorded more frequently when participants were on Gla-300 (69.1%; 38 nights) than when on Gla-100 (31 nights; 54.4%). Moreover, a reduction in the cumulative frequency of individuals to present with hypoglycemic (19.30%, 11 nights, vs. 16.40%, 9 nights) or hyperglycemic (26.30%, 15 nights, vs. 14.50%, 8 nights) glucose levels at 00:00 AM on CGM nights was also observed when patients switched from Gla-100 to Gla-300 for basal insulin therapy.

### 3.4. Assessment of Intranight Glucose Variability by CGM

**Figure 2:**
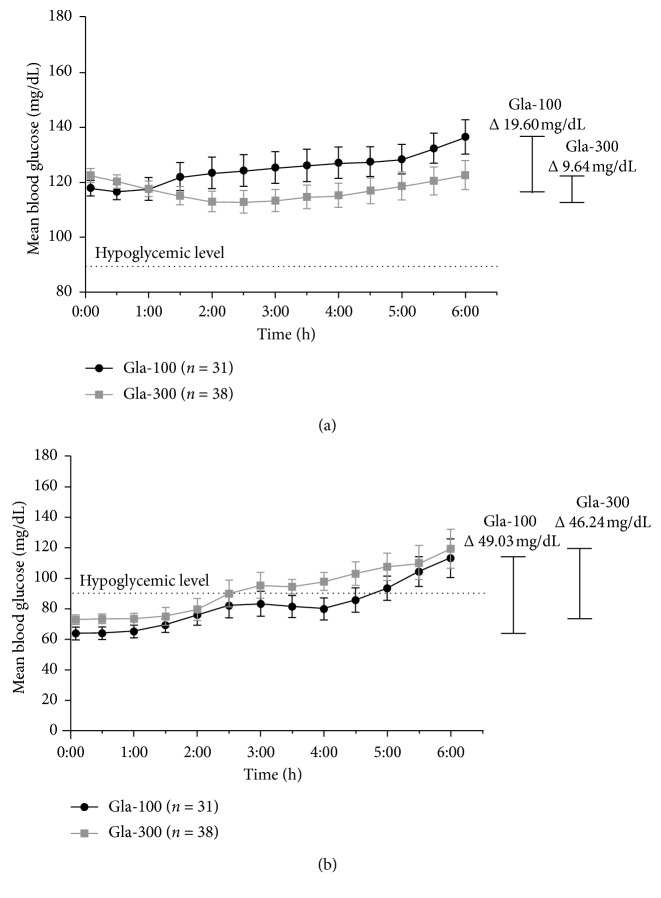
Mean nocturnal glucose profiles (00:00–6:00 AM) displayed by patients when glucose levels were (a) 90–160 mg/dL and (b) <90 mg/dL at 00:00 AM. Individual data points represent the mean blood glucose level at 30 min intervals (mean ± SEM).

Assessment of intranight glucose variability during the interval of 00:00–6:00 AM associated with Gla-300 and Gla-100 treatments was performed by evaluating the mean 6-hour nocturnal glucose profiles of each treatment phase for variances.  Figure 2 displays the mean nocturnal glucose profile experienced by participants during each treatment phase when their nocturnal glucose levels were measured to be between 90 and 160 mg/dL (Figure 2(a)) and <90 mg/dL (Figure 2(b)) at 00:00 AM. Individual data points represent the mean glucose level per 30 min interval.

### 3.5. Intranight Glycemic Variability on Normal Nocturnal Glucose Levels (90–160 Mg/dL)

The mean intranight nocturnal glucose profile appears to be flatter during the Gla-300 treatment period than the Gla-100 treatment period when normoglycemia was measured by CGM at 00:00AM, suggestive of Gla-300 providing a steadier distribution of intranight glucose-lowering activity than Gla-100. Indeed, the mean SD of nocturnal glucose levels from 00:00–6:00 AM was 29.87 ± 4.68 mg/dL versus 25.52 ± 5.34 mg/dL (*p* < 0.0001) when on Gla-100 and Gla-300 therapy, respectively. The within-night CV during the same time frame was 23.73 ± 3.2% versus 21.87 ± 4.39% (*p* < 0.0001) for Gla-100 and Gla-300 therapy, respectively. The mean intranight difference between maximal and nadir nocturnal glucose levels during the Gla-300 treatment phase was 2 folds lower (Δ9.64 mg/dL) than the Gla-100 treatment phase (Δ19.60 mg/dL).

### 3.6. Intranight Glycemic Variability on Low Nocturnal Glucose Levels (<90 Mg/dL)

Glucose levels were observed to elevate towards normal glucose levels during both the Gla-100 and Gla-300 treatment phases on nights; nocturnal glucose levels were measured to be <90 mg/dL at 00:00 AM (Figure 2(b)). Notably, the gradient of the Gla-300 glucose curve on these nights was more constant than that of the Gla-100 glucose curve, an indication of Gla-300's superior glycemic variability control. Overall, the mean duration of time spent in nocturnal hypoglycemia during the Gla-300 treatment period was approximately 2.1 times less (130 min vs 270 min) than that in the Gla-100 treatment phase. The mean within-night SD of nocturnal glucose levels was 22.38 ± 7.34 mg/dL versus 21.07 ± 7.89 mg/dL (*p* < 0.2157), and mean within-night CV of nocturnal glucose levels was 27.42 ± 4.47% versus 22.02 ± 5.86% (*p* < 0.0001) during Gla-100 and Gla-300 treatment phases, respectively. The mean intranight difference between the maximum and minimum glucose levels during the Gla-300 treatment phase (Δ46.24 mg/dL) was found to be numerically lower than that during the Gla-100 treatment phase (Δ49.9 mg/dL).

## 4. Discussion

To our knowledge, this study represents the first case-series report from Taiwan that evaluates the real-world clinical benefits of switching Taiwanese diabetes patients from Gla-100 to Gla-300 for basal insulin therapy with regard to glucose variability minimization and nocturnal hypoglycemia reduction by means of CGM.

Glucose variability is associated with the increased risk of severe hypoglycemia [20, 21]. In the present study, nocturnal glucose levels (00:00–6:00 AM) during the Gla-300 treatment period were observed to be comparable to that observed during the Gla-100 treatment period, with a trend for the measured parameters of nocturnal glycemic variability to be lower when Gla-300 was used for basal insulin therapy. Given that there was a trend for Gla-300 to improve overall nocturnal glycemic variability more than Gla-100, it is possible that this was in part due to Gla-300 providing greater within-night glycemic variability control over Gla-100. Indeed, exploratory analysis of the mean 6-hour nocturnal glucose profiles generated during each treatment phase revealed that Gla-300 provided significantly more stable within-night control of nocturnal blood glucose levels than Gla-100, aligning well with the more constant PK/PD profiles of Gla-300 over Gla-100 described in the literature [10]. Future studies using a larger study population over an extended period of time should be conducted to further confirm this relationship.

A worrisome safety concern associated with fluctuating nighttime glucose levels is nocturnal hypoglycemia as patients are often unaware of the occurrences of these events due to suppressed sympathoadrenal signals during sleep [4, 5]. The lack of unawareness for the need for self-treatment during sleep can lead to a dangerous cycle of self-exacerbating nocturnal hypoglycemia resulting in restlessness during sleep, chronic fatigue, and cognitive impairment, perpetuating poor management of glucose levels [4, 5]. In the EDITION JP 1 and EDITION JP 2 trials, respectively, Gla-300 has been demonstrated to reduce the frequency of confirmed or severe nocturnal hypoglycemia over Gla-100 in T1DM and T2DM patients with prior insulin experience over a 6-month study period [16, 18]. This reduced frequency of nocturnal hypoglycemia has been attributed to greater control of glycemic variability associated with Gla-300 over Gla-100 through CGMS studies [11–13]. In line with this reported relationship, a modest 2.9% numerical decrease in the detection of nocturnal hypoglycemia at 00:00 AM during the Gla-300 treatment period over the Gla-100 treatment phase is observed in parallel with the trend for Gla-300 to provide greater night-to-night glycemic variability control in the present CGM study. Cumulatively, these results highlight that prescribing Gla-300 over Gla-100 may provide greater protection against the protection against nocturnal hypoglycemia in Taiwan clinical practice.

A limitation of the present study is the small and heterogeneous sample size used which may in part have limited the ability of some of the trends observed to reach adequate statistical power. Another limitation of the study was the short treatment period investigated which may not have allowed for an adequate full representation of the clinical benefits of switching patients to Gla-300 from Gla-100 in nocturnal glycemic variability control to be observed. Nonetheless, this study provides critical information to Taiwanese clinicians on the potential benefits of Gla-300 over Gla-100 in the real world as diet decisions, time of dosing, and length of sleep were left to the discretion of individuals in this study.

## 5. Conclusion

This case-series study demonstrates that Gla-300 is comparable to Gla-100 in providing tight glycemic control and has the potential to reduce the frequency of nocturnal hypoglycemia over Gla-100. Notably, exploratory analysis in this study demonstrates that several measures of intranight glycemic variability are statistically lower when Gla-300 is used in place of Gla-100 for basal insulin therapy.

## Figures and Tables

**Table 1:** Mean nocturnal glucose level (00:00 AM) and parameters of glycemic variability (00:00–6:00 AM) before and after switching from Gla-100 to Gla-300 for basal insulin therapy.

Parameters	Treatment		
	Gla-100	Gla-300	*p* value
Mean nocturnal glucose level (mg/dL)	126.1 ± 35.11	121 ± 31.23	0.421
SD (mg/dL)	16.21 ± 11.04	15.18 ± 9.76	0.605
CV (%)	13.34 ± 9.95	12.69 ± 8.02	0.703
Mean amplitude of glucose excursions	55.58 ± 35.39	52.09 ± 31.09	0.581

Data are given as mean ± SD.

## Data Availability

The continuous glucose monitoring data used to support the findings of this study are available from the corresponding author upon request.
